# Lisfranc Injuries: Latest Updates on Diagnostics and Management

**DOI:** 10.1155/tsm2/3933956

**Published:** 2026-01-07

**Authors:** Ahmad Hammad, Yasser Ahmad, John Abdelnour

**Affiliations:** ^1^ Department of Orthopedics Surgery, American University of Beirut Medical Center, Beirut, Lebanon, aubmc.org.lb

**Keywords:** arthrodesis, bridge plating, Lisfranc injury, orthopedics surgery, surgery, tarsometatarsal joint

## Abstract

The Lisfranc ligamentous complex is the principal stabilizer of the tarsometatarsal joint and functions as the midfoot keystone. Injuries often follow an axial or rotational load applied to a plantarflexed foot, producing ligamentous disruption or, in severe cases, fracture–dislocation of the tarsometatarsal complex. Up to one‐third of Lisfranc injuries are initially missed, particularly in low‐energy mechanisms or polytrauma settings. Weight‐bearing radiographs are essential for detecting subtle injuries and uncovering diastasis between the medial cuneiform and the second metatarsal. The Myerson classification categorizes injuries based on joint congruity, the direction of displacement, and extent of involvement. The decision to pursue conservative or surgical treatment depends on the Lisfranc ligament stability and displacement. Nonoperative management is appropriate only in nondisplaced injuries; delayed treatment can result in persistent midfoot pain, arch collapse, post‐traumatic arthritis, and diminished function. Surgical techniques include open reduction and internal fixation, primary arthrodesis, bridge plating, suture button fixation, and percutaneous approaches. Lateral column injuries involving the fourth and fifth tarsometatarsal joints are advised to be treated with K‐wire fixation. Anatomic alignment is the strongest predictor of successful recovery and return to activity. Residual displacement > 2 mm is associated with inferior outcomes and significantly reduced return‐to‐play rates, particularly in athletes who can have lasting effects even with successful fixation and may not reach preinjury performance levels. Optimal management is yet to be determined, and inadequate fixation increases poor outcomes, underscoring the importance of early recognition, precise reduction, and appropriate fixation strategy. This study is novel and integrates recent evidence including diagnostic and prognostic utility of weight‐bearing, the clinical outcomes and biomechanics of treatment approaches including flexible fixation constructs such as suture button systems, and postoperative outcomes including gait analysis, return‐to‐play, and athletic performance outcomes.

## 1. Introduction and Anatomy

Anatomical vulnerability is due to the absence of a transverse intermetatarsal ligament between the first and second metatarsals. The Lisfranc ligamentous complex (LLC) originates from the plantar medial cuneiform (C1) and inserts into the medial base of the second metatarsal (M2) and is the principal stabilizer of the tarsometatarsal (TMT) joint, particularly the articulation between the C1 and the base of M2 [1]. This articulation forms a mortise configuration that locks the midfoot and preserves transverse arch integrity [1, 2]. This structural arrangement prevents lateral or medial translation and functions as the keystone of the midfoot [2, 3].

The LLC is composed of three primary ligaments: the dorsal (DLL), interosseous (ILL) commonly referred to as the Lisfranc ligament, and plantar (PLL) ligaments [2, 4]. Among these, the ILL is the thickest and most biomechanically robust, with the largest M2 insertional area, greatest stiffness, and highest resistance to failure load [2], such as injury to the ILL particularly poses concerns for midfoot stability. The Tricolumn theory divides the midfoot into medial, middle, and lateral columns, which influences fixation strategy. The central column including the second TMT joint serves as the keystone of the midfoot arch and is mainly susceptible to instability [1].

The structural gap at the Lisfranc region contributes to the high susceptibility of this articulation to both direct trauma and indirect loading mechanisms. Additionally, the second TMT joint is recessed relative to adjacent articulations, further concentrating mechanical stress in this area [5]. Variants such as a shallow mortise or short second metatarsal are associated with increased risk of injury [1].

Injuries to the Lisfranc joint are categorized as ligamentous or bony; ligamentous injuries involve soft tissue disruption without associated fractures and can be more difficult to detect clinically and radiographically [4], whereas bony Lisfranc injuries involve avulsion or intra‐articular fractures, typically visible on imaging [3, 5]. Subtle or incomplete ligamentous injuries are particularly prone to underdiagnosis and being initially missed [4, 6]. Delayed identification of these injuries may result in chronic instability, post‐traumatic arthritis, and functional impairment; thus, isolated dislocations without fractures may actually have a worse outcome than injuries with fractures. Hence, early detection of subtle injuries and anatomic reduction are necessary for optimal outcomes [7]. The aim of treatment is anatomic reduction, followed by fixation or arthrodesis; however, consensus is yet to be achieved on the superior approach [7, 8].

The typical dislocation pattern is dorsal, attributed to the relative weakness of the dorsal ligaments compared to the stronger plantar ligaments [1, 2]. This loss of restraint allows dorsal translation of the metatarsal bases, compromising the midfoot arch and resulting in deformity if not anatomically reduced. The aim of this paper was to review Lisfranc ligament injuries with a focus on etiology, classification, and emerging evidence in diagnostic measures, treatment techniques and outcomes, and complications. This review is different by integrating recent evidence including diagnostic and prognostic utility of weight‐bearing CT (WBCT), the clinical outcomes and biomechanics of flexible fixation constructs such as suture button systems, and return‐to‐play and athletic performance outcomes, which were not comprehensively synthesized in prior reviews.

## 2. Methodology

We conducted a quantitative synthesis of all studies comparing different surgical treatment of meniscal tear, according to the Preferred Reporting Items for Systematic Reviews and Meta‐Analyses (PRISMA) guidelines with a PRISMA checklist and algorithm. A literature review was performed using Cochrane Library, PubMed, MEDLINE, and Scopus, and no restrictions were made regarding language, publication status, and clinical study design covering publications from 2010 to 2025. Supplemental data were identified through a random search on Google and Google Scholar. This is a retrospective narrative review article that did not require approval from the institutional review board.

The search strategy used the following medical subject headings (MeSH) and terms: “Lisfranc injury”, “tarsometatarsal joint”, “arthrodesis”, “bridge plating”, and “return to play”. We included English‐language clinical studies, reviews, and biomechanical analyses with emphasis on those published in the past 5 years. Reference screening of cited bibliographies was used to identify additional key studies. This addition enhances transparency and allows readers to assess the scope of evidence considered.

**Figure 1:**
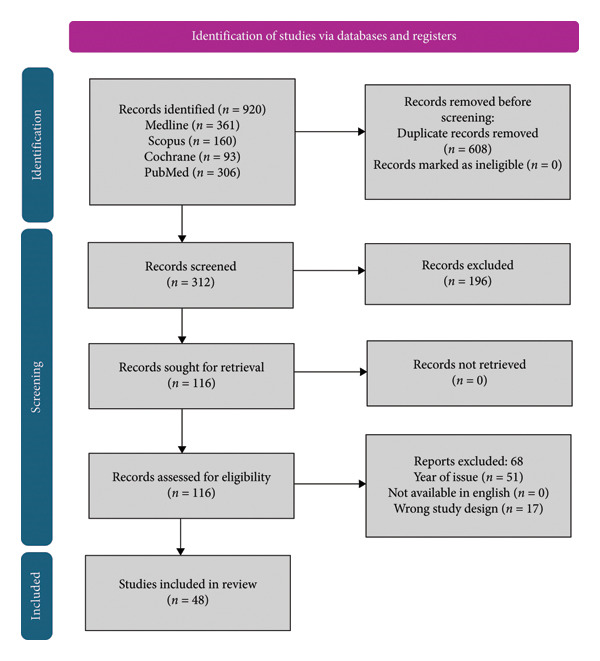
Prisma flow diagram.

We systematically reviewed studies if they met the inclusion criteria: English‐language studies, level 1 to 4 studies comparative and observational studies, Lisfranc injury with no restriction to age (above 18) and sex. Exclusion criteria included case reports, animal studies, articles not in the English language, and experts’ opinions. Initial search resulted in 920 records identified for screening; however, after duplicates and studies that did not meet the inclusion criteria per study design and date of publication year were excluded, 116 articles were assessed, of which 48 were included. The search algorithm according to the PRISMA guidelines is shown in Figure 1.

## 3. Incidence, Etiology, and Biomechanics

Although traditionally considered rare, Lisfranc injuries likely occur more frequently than reported. While the general incidence is around 0.2% of all fractures [1, 4], newer studies using advanced imaging suggest a higher true prevalence [5, 9]. Up to 30% of cases are initially missed, particularly in low‐energy mechanisms or polytrauma settings [6, 10, 11].

The classic mechanism involves axial or rotational load applied to a plantarflexed foot, leading to dorsal ligament failure or fracture at the metatarsal base [1, 3]. Other mechanisms include twisting of the forefoot or crush injuries. High‐energy trauma, such as motor vehicle accidents, falls, or crush injuries, can produce extensive bony and soft tissue damage, often involving multiple TMT joints [1]. In contrast, low‐energy injuries are increasingly observed in sports, especially football, rugby, and soccer [4, 12]. Football players, particularly those in offensive positions, are at increased risk, with Lisfranc injuries affecting up to 20% of collegiate players annually [12]. Despite growing recognition, many sports‐related injuries remain underdiagnosed due to subtle presentations and limitations in early imaging.

Recent biomechanical investigations have quantified the mechanical properties of LLC demonstrating that ILL exhibits the highest stiffness (380–450 N/mm) and failure load (200–300 N) compared to the dorsal and plantar components, confirming its role as the primary stabilizer of the TMT joint [13, 14]. These quantitative data support fixation strategies that restore the natural stiffness ratio between the dorsal and plantar ligaments to prevent midfoot over‐constraining. Cadaveric studies using strain‐gauge and motion‐analysis techniques have shown that the second TMT joint bears up to 40%–45% of midfoot load transmission during stance [15], while in vivo WBCT finite element models reveal asymmetric load transfer and dorsal stress concentration at the C1‐M2 interface under physiologic conditions [16]. This discrepancy between static cadaveric data and dynamic WBCT‐based modeling underscores the need for fixation constructs that mimic physiologic micromotion rather than rigid constraint, particularly in athletic populations. Incorporating these biomechanical insights into surgical planning can improve construct design and optimize fixation angles to better reproduce normal load‐sharing mechanics of the Lisfranc complex.

## 4. Diagnosis: Imaging and Physical Examination

Accurate diagnosis of Lisfranc injuries requires a combination of clinical suspicion, targeted physical examination, and appropriate imaging. Up to 20% of injuries are missed on initial evaluation, particularly when non‐weight‐bearing (NWB) imaging is used in subtle or ligamentous cases [1, 4].

Following trauma, patients typically present with midfoot pain, swelling, and difficulty bearing weight. Plantar ecchymosis is considered pathognomonic [1]. Physical examination maneuvers such as the “piano key test” (mobilizing the metatarsal head while stabilizing the midfoot), abduction stress testing, and assessment of the medial arch gap may reveal instability [17, 18]. A positive single‐limb toe rise test may indicate functional instability, while high‐energy injuries warrant vigilance for compartment syndrome.

Plain radiographs remain the first‐line imaging modality with standard views include anteroposterior (AP), lateral, and oblique projections. However, weight‐bearing radiographs are essential for detecting subtle injuries and uncovering diastasis between the medial cuneiform and the second metatarsal [4, 9]. Even in cases where NWB images appear normal, weight‐bearing can reveal pathologic widening or malalignment. Classic signs include a “fleck sign” (avulsion between C1 and M2) and malalignment of the medial borders of the second metatarsal and middle cuneiform on AP view. Diagnostic thresholds on radiographs have been proposed, and as compared with the contralateral, uninjured foot under equivalent loading conditions, including a first‐to‐second metatarsal (M1–M2) diastasis > 4 mm NWB or > 5 mm weight‐bearing, and a medial cuneiform to second metatarsal (C1–M2) gap > 3 mm NWB or > 5 mm weight‐bearing [4, 19].

When radiographs are inconclusive, computed tomography (CT) is recommended when suspicion is high, especially in high‐energy trauma. CT identifies occult fractures and subtle subluxations not visible on X‐ray [1, 4]. However, conventional CT lacks dynamic assessment under physiologic load. WBCT has emerged as a more sensitive tool for identifying subtle Lisfranc instability. It offers superior assessment of joint morphology and dorsal ligament disruption under load‐bearing conditions as compared to non‐WBCT and MRI [9, 20]. WBCT has demonstrated superiority in detecting dorsal ligament injuries and subtle instability, particularly in cases with minor symptoms that may appear normal on conventional CT or MRI [9]. WBCT provides superior agreement (*K* = 0.78) to conventional CT (*k* = 0.61) and MRI (*k* = 0.67) for detecting subtle Lisfranc instability [21].

MRI is useful for evaluating soft tissue and ligamentous injuries, especially in cases of chronic pain or isolated ligament disruption with negative radiographs; however, limitations include cost, availability, and lack of load‐bearing capability [22]. MRI has demonstrated high diagnostic performance with sensitivity ranging from 94% to 97% and specificity between 75% and 88% when compared with intraoperative findings highlighting MRI’s value in detecting purely ligamentous disruptions that may not be visible on radiographs or CT particularly in early or subtle injury patterns [10].

Despite its diagnostic advantages, WBCT remains limited in availability and cost‐effectiveness, particularly outside tertiary care and sports medicine centers [16]. The technology requires specialized hardware, increased image processing time, and higher per‐scan costs compared with standard CT or MRI, which currently restrict its routine use. Therefore, while WBCT enhances diagnostic precision, its integration into everyday practice must balance clinical utility against accessibility and resource allocation. Ultrasound is not recommended as a primary diagnostic tool due to operator dependence and inability to visualize deeper ligamentous structures reliably [4].

## 5. Classification

Several systems have been proposed to classify Lisfranc injuries with distinct clinical utility, but none is universally adopted for both prognostic and treatment purposes. The most widely used is the Myerson classification, which categorizes injuries based on joint congruity, the direction of displacement, and extent of involvement. Originally developed in 1986, it includes three main types (A: total incongruity, B: partial incongruity, C: divergent) that were modified to include subtle injuries. The updated system adds Type D injuries which are nondisplaced and particularly rely on assessment of weight‐bearing stability, as subtle displacement may only become evident under physiologic load; they are subdivided into D1 (stable, nonoperative) and D2 (unstable, operative), with D2L indicating purely ligamentous injuries and D2B denoting bony avulsions [1, 18].

For athletic injuries, the Nunley and Vertullo classification attempts to stage injuries based on physical exam, weight‐bearing radiographs, and bone scintigraphy. While helpful in identifying subtle sprains, it has not achieved widespread use, in part due to the availability of MRI for more precise diagnosis [1, 23]. Another emerging system is the CT‐based classification by Schepers and Rammelt, which divides Lisfranc injuries by column (medial, central, lateral) and fracture type (avulsion, simple, comminuted). Although this approach allows detailed anatomic categorization, its complexity and lack of integration of displacement make it difficult to apply routinely [24].

Table 1 presents a comparison and summary of the main Lisfranc classification systems, their defining features, imaging basis, and practical utility. While each system contributes specific strengths, no current classification reliably guides treatment and prognosis across all injury types. However, Myerson’s remains the most clinically used due to its high interobserver reliability and relevance to surgical planning [1]. Recent studies have proposed the integration of WBCT‐derived quantitative metrics such as dorsal cuneiform—metatarsal subluxation angles, sagittal plane displacement ratios, and 3D diastasis indices—to enhance injury grading and surgical planning [16, 25]. These models aim to provide objective, reproducible thresholds for defining instability and treatment indications, potentially complementing or refining traditional radiographic classifications. While promising, further validation and outcome correlation studies are required before these quantitative WBCT‐based classifications can be adopted in clinical algorithms.

**Table 1:** Comparative summary of Lisfranc injury classification systems.

System	Basis/imaging modality	Key criteria/features	Clinical utility/limitations
Myerson (1986, modified 2015)	Radiographic/CT	Type A: total incongruity; Type B: partial incongruity (medial or lateral); Type C: divergent; Type D1 (stable, nondisplaced), D2 (unstable, displaced; D2L = ligamentous, D2B = bony)	Most widely used; guides surgical approach; high interobserver reliability; limited prognostic value
Nunley–Vertullo (2002)	Clinical + weight‐bearing radiographs/bone scintigraphy	Stage I: pain, no diastasis; Stage II: diastasis without arch collapse; Stage III: diastasis with loss of arch height	Useful for athletic, low‐energy injuries; early detection of subtle sprains; limited use with modern MRI availability
Schepers–Rammelt (2020)	CT‐based	Column‐based: medial, central, lateral; subdivided by fracture pattern (avulsion, simple, comminuted)	Provides detailed anatomic mapping; aids preoperative planning; complex, limited correlation with stability
Emerging WBCT‐based models (2023–2025)	WBCT/3D reconstruction	Quantitative metrics: dorsal subluxation angle (> 4°), sagittal translation ratio (> 10%), C1–M2 diastasis index (> 3 mm under load)	Improves objectivity and reproducibility; still under validation; potential to refine traditional systems

*Note:* C1: medial cuneiform; 3D, three dimensions; M2: second metatarsal; WBCT, weight‐bearing CT.

Abbreviation: CT, computed tomography.

## 6. Management Options

The decision to pursue conservative or surgical treatment in Lisfranc injuries depends primarily on the presence of instability and displacement. Nonoperative management is appropriate only in nondisplaced injuries with < 2 mm of diastasis, typically confirmed on weight‐bearing or CT imaging [1, 24]. Such injuries are often midfoot sprains or low‐grade avulsion fractures and can be treated with 4–6 weeks of NWB casting followed by progressive return to weight‐bearing [5].

In contrast, displaced injuries or those with instability require surgical intervention. Displacement of ≥ 2 mm between the C1 and base M2 on imaging is widely accepted as an indication for operative management [24]. Further indications may include more than 15° of talo‐metatarsal angulation in the sagittal plane since it indicates unstable injury [26]. Operative fixation is also advised when symptoms persist or joint malalignment is evident on advanced imaging, even in the absence of clear displacement on plain films [4]. Despite the importance of surgical decision‐making, a systematic review found that most studies failed to provide clear operative indications, highlighting a need for standardized criteria [27].

### 6.1. Surgery

Surgical techniques include open reduction and internal fixation (ORIF), primary arthrodesis, bridge plating, flexible fixation with suture button constructs, and percutaneous approaches. The aim of surgery is to achieve anatomic reduction, which is consistently linked to better outcomes regardless of fixation type [28, 29].

ORIF is most commonly performed using transarticular screws, particularly from C1 to the base of M2. This construct, often termed the “home run screw,” restores alignment of the keystone articulation and stabilizes the medial and middle columns [1]. A partially threaded screw may be used to achieve compression, although care must be taken to avoid overcompression by excessive tightening of transarticular screws, which may cause articular step‐off or metatarsal base distraction, leading to subluxation. Alternatively, some surgeons employ a reverse orientation, inserting the screw from M2 to C1, a method described by Panchbhavi et al. [30]. This direction offers certain advantages: the threaded portion of the screw lies within the larger cuneiform bone, potentially reducing the risk of distraction at the metatarsal base, and if hardware removal becomes necessary, the screw head is more easily accessed at the metatarsal base [1]. Supplemental fixation may include screws placed between C1 and C2 or from the first metatarsal to the medial cuneiform.

Bridge plating is a joint‐sparing alternative to screw fixation. Plates are applied dorsally across the joint without violating the articular surface. Biomechanically, bridge plates offer comparable stability to screws and have been associated with better functional scores, lower rates of post‐traumatic arthritis, and reduced hardware failure [29, 31]. However, excessive soft tissue dissection and the potential for cartilage compression with repeated passes are concerning [31]. Primary arthrodesis is increasingly used, particularly in chronic or purely ligamentous injuries, and shows better functional outcomes in few studies compared to ORIF, along with lower rates of hardware removal [6]. However, arthrodesis sacrifices joint motion and may be less desirable in younger or athletic patients. Notably, hardware removal is often required following transarticular screw fixation due to articular surface violation, which increases the risk of chondral damage and post‐traumatic arthritis [7, 26].

Flexible fixation with suture button constructs has gained attraction as a joint‐preserving method. It avoids rigid transarticular compression, allows physiological micromotion, and is associated with excellent return to sport (RTP) rates and low complication rates [28, 32]. In athletes, suture button fixation has been associated with shorter return‐to‐play times and fewer secondary procedures compared to ORIF or arthrodesis. However, most studies included small cohorts (ranging from 45 to 186 patients), reflecting rarity and heterogeneity. Percutaneous fixation is reserved for cases with preserved soft tissue envelope and less comminution. It is less invasive and offers good outcomes in Myerson B injuries, provided anatomic reduction is achieved [3]. In delayed presentations, arthroscopically assisted debridement and reduction, followed by percutaneous fixation, has been proposed when open reduction is contraindicated [3]. Percutaneous and arthroscopically assisted approaches, while less invasive and cosmetically favorable, are technically demanding and risk incomplete reduction due to limited visualization, especially in multicolumn injuries. Their long‐term durability and comparative outcomes remain under investigation, with current studies limited to short‐term case series [33].

Comparative outcome data provide further insight into the relative performance of different fixation strategies. Anatomic reduction remains the strongest predictor of good outcomes. In terms of postoperative scores: mean AOFAS scores (ORIF 82–86; arthrodesis 87–91; bridge plating 89‐90; suture button fixation 92–95), hardware removal rates (ORIF 45%–60%; arthrodesis 20%–25%; suture button < 10%), and mean return‐to‐play times (ORIF 24–28 weeks; arthrodesis 22–25 weeks; suture button 16–18 weeks).

### 6.2. Lateral Column Fixation

The lateral column of the foot serves a more flexible, mobile function compared to the medial and middle columns and comprises the fourth and fifth metatarsals and the cuboid. Following injury, transarticular Kirschner wires (K‐wires) are the preferred method of stabilization for lateral column; typically removed at 6 weeks to preserve mobility and reduce long‐term stiffness [1, 29]. Rigid fixation with screws in this region may disrupt normal foot mechanics and decrease physiological motion due to the risk of overconstraining a joint that naturally accommodates more flexibility during gait. [1]. Over‐rigid fixation leads to altered biomechanics and poor functional outcomes particularly in high‐demand patients.

### 6.3. Rehabilitation

Following operative fixation of Lisfranc injuries, patients are typically placed in a cast or boot with NWB for 6–8 weeks, depending on the fixation type and injury severity [1]. Progressive weight‐bearing is initiated, often beginning with a walking boot and transitioning to regular footwear by 12 weeks, then physical therapy is introduced to address gait normalization, edema control, and joint mobility. Table 2 summarizes the proposed evidence‐based postoperative rehabilitation that follows a phase‐based loading anchored to objective criteria rather than fixed dates alone [34]. After an initial 6–8 weeks of NWB for most rigid constructs (earlier protected loading may be used with joint‐preserving or flexible constructs at the surgeon’s discretion), patients progress to partial weight‐bearing (PWB) in a boot with incremental loading (e.g., 25% body weight increases weekly) contingent on: pain ≤ 3/10 during/after activity, minimal swelling, intact alignment on surveillance imaging, and ability to perform 10 pain‐free heel raises [35]. Transition to full weight‐bearing (FWB) and a shoe with a stiff insert generally occurs by 10–12 weeks, followed by impact reintroduction at ∼12–16 weeks and field/court drills at ∼16–20 weeks, assuming strength, balance, and hop symmetry thresholds are met. Athlete‐focused programs should emphasize proprioception, midfoot control, calf‐intrinsic synergy, and gait retraining to restore a normal forefoot rocker and push‐off mechanics, which are commonly impaired after Lisfranc injury [36].

**Table 2:** Rehabilitation phases, objective criteria, and milestones after Lisfranc repair.

Phase/typical timeframe	Primary goals	Key interventions	Progression criteria	Milestones
I. Protection/NWB 0—6–8 weeks	Protect fixation; edema/pain control; maintain proximal conditioning	NWB in cast/boot; edema control; isometrics (hip/knee/core); gentle ankle ROM if permitted	Pain ≤ 3/10 at rest; controlled swelling; incision healed	Radiographic check (alignment maintained); lateral K‐wires removed ≈6 weeks if used
II. PWB ⟶ FWB (Boot) ∼6—10–12) weeks	Gradual load tolerance; normalize gait in boot	Begin PWB (≈25% BW weekly increases); stationary bike; pool; ankle/foot intrinsic activation; balance in boot	Pain ≤ 3/10 during/after loading; minimal swelling next day; 10 pain‐free bilateral heel raises	FWB in boot; transition to stiff‐insert shoe once pain‐free gait achieved
III. Strength/Control (Shoe) ∼10—16 weeks	Restore strength, proprioception, midfoot control	Intrinsic foot + calf strength; closed‐chain balance; gait retraining (midfoot rocker, push‐off); anti‐gravity treadmill as needed	≥ 80–85% strength symmetry; pain‐free 30 single‐leg calf raises; normalized walking gait	Initiate linear jogging; low‐amplitude cutting drills
IV. Plyo/Change‐of‐Direction ∼16—20 + weeks	Power, agility, sport‐specific skills	Plyometrics; COD drills; progressive forefoot loading; return‐to‐practice ramps	≥ 90% hop/Y‐Balance symmetry; no effusion or next‐day pain/swelling	Noncontact team practice; controlled scrimmage
V. RTP Individualized (often 20—28 + weeks; elite athletes sometimes earlier with flexible fixation)	Full performance restoration	Sport‐specific conditioning and exposure	FAAM‐Sports ≥ 85–90; athlete/coach/medical consensus	Full competition return

Abbreviations: BW, body weight; COD, change of direction; FAAM, Foot and Ankle Ability Measure; FWB, full weight‐bearing; NWB, non–weight bearing; PWB, partial weight‐bearing; ROM, range of motion.

For patients treated with K‐wires, removal is generally scheduled at 6 weeks to preserve lateral column flexibility. In cases involving screws or plates, hardware removal is variably practiced; while some advocate for routine removal to restore midfoot motion, others opt for retention unless symptomatic. Pure ligamentous injuries may be kept fixed and off weight‐bearing for longer periods of time. Long‐term follow‐up is essential to monitor for complications such as post‐traumatic arthritis, hardware irritation, or residual instability [37].

## 7. Outcomes: Return to Ambulation and Sports

Functional outcomes after Lisfranc injury depend primarily on the quality of reduction, regardless of fixation type. Anatomic alignment is the strongest predictor of successful recovery and return to activity [1, 12, 29]. Following surgical treatment, weight‐bearing ambulation typically resumes after 6–8 weeks of immobilization, with most patients returning to regular footwear by 3 months [1]. Physical therapy aids in restoring gait and reducing edema. Full recovery can take 4–6 months or longer in high‐energy injuries.

Instrumented and observational gait studies show reduced walking speed, diminished midfoot motion, and altered push‐off after Lisfranc injury; targeted interventions such as foot intrinsic strengthening, tibialis posterior/peroneal co‐contraction, ankle plantarflexor power, and progressive forefoot loading drills are recommended to normalize the midfoot rocker [38]. RTP should be criteria‐based: pain‐free sport‐specific drills, < 10% side‐to‐side edema difference, ≥ 90% symmetry on single‐leg hop and Y‐Balance tests, ≥ 90% plantarflexion strength symmetry (isokinetic or handheld dynamometry), and functional confidence/outcome thresholds (e.g., FAAM‐Sports ≥ 85–90) [36]. In athlete series and systematic syntheses, RTP commonly occurs between 16 and 28 weeks, with faster timelines reported for flexible/suture‐button constructs when anatomic alignment is maintained [39].

Nonoperative treatment can be effective for nondisplaced injuries. Pain and functional scores are comparable to ORIF, and RTP rates reach 93% provided instability is ruled out [5, 40]. ORIF remains standard but is associated with higher rates of hardware irritation and potential cartilage damage. Some studies report worse functional scores and slower RTP compared to arthrodesis or suture button fixation; nonetheless, functional and pain scores are satisfactory on the long term [6, 29, 41]. Primary arthrodesis offers superior scores compared to ORIF, particularly in chronic or purely ligamentous injuries, and also carries lower hardware removal rates but sacrifices joint motion [6, 33]. Suture button fixation shows the shortest return‐to‐play time (∼17 weeks) and highest RTP rate (100%) in small athlete cohorts, though long‐term durability data (follow‐up ≥ 24 months) remain limited [42]; no hardware failures or secondary surgeries were reported, and athletes maintained alignment with minimal pain [28, 32].

Across treatment methods, RTP rates range from 86% to 100%, with mean RTP time between 16 and 29 weeks [32, 40]. RTP varies by activity; in European soccer and rugby players, the average return time was 25 weeks [43], while NFL athletes averaged 11 months, often with performance decline in the first post‐injury season [44]. However, performance decline in the first season is common, with reduced games played, slower recovery, and decreased likelihood of being drafted, especially in players with > 2 mm residual displacement [12]. Despite favorable RTP statistics, Lisfranc injuries can have lasting effects. Even athletes with successful fixation may not reach preinjury performance levels for 1–2 seasons. Long‐term studies revealed persistent midfoot stiffness, especially after rigid fixation or missed diagnosis [12].

## 8. Complications of Delayed, Missed, or Poor Treatment & Surgical Complications

A missed diagnosis or inadequate fixation increases the likelihood of poor outcomes, underscoring the importance of early recognition, precise reduction, and appropriate fixation strategy. Residual displacement > 2 mm is associated with inferior outcomes and significantly reduced return‐to‐play rates, particularly in athletes [12]. Even with fixation, poor reduction quality is a major predictor of long‐term dysfunction. Hence, despite adequate fixation, post‐traumatic and postoperative arthritis and mal‐reduction may require secondary fusion of the first 3 tarso‐metatarsal joints (excluding the fourth and fifth).

### 8.1. Missed Diagnosis & Delayed Treatment

Lisfranc injuries are frequently underdiagnosed, and up to one‐third are missed on initial presentation, particularly in low‐energy or purely ligamentous cases [1, 6]. Delayed or inadequate treatment can result in persistent midfoot pain, arch collapse, post‐traumatic arthritis, and diminished function [45]. Furthermore, it may lead to foot deformities such as planus, planovalgus, cavus, and forefoot abduction or adduction [26]. Late surgical intervention beyond 6 weeks is technically challenging due to ligament scarring, soft tissue changes, and joint malalignment because the tibialis anterior tendon & peroneus longus tendons displace between the middle and medial cuneiform & hinder reduction [2].

### 8.2. Surgery

Complications remain closely tied to fixation method as summarized in Table 3. Transarticular screw fixation is associated with the highest rate of hardware irritation (45%–60%) and routine removal due to articular cartilage violation [29, 31, 46]. Bridge plating reduces hardware irritation (20%–30%) and post‐traumatic arthritis incidence by avoiding intra‐articular penetration [26]. Primary arthrodesis demonstrates fewer reoperations for pain (15%–25%) but may lead to adjacent joint overload in younger, active patients. Flexible suture button fixation carries the lowest rate of hardware removal (< 10%) and postoperative arthritis while preserving physiological joint motion [14, 28, 32, 42]. Infection, nerve irritation, and secondary arthrodesis occur infrequently (< 5%) across modalities when anatomic reduction is achieved.

**Table 3:** Complication rates by fixation type.

Fixation method	Hardware irritation/removal (%)	Post‐traumatic arthritis (%)	Nerve injury (%)	Reoperation for pain/nonunion (%)	Notes
ORIF (screws)	45–60	25–35	5–10	10–15	High irritation; cartilage damage from transarticular screws
Bridge Plating	20–30	10–15	3–5	8–10	Lower arthritis rate; preserves cartilage; requires soft‐tissue dissection
Primary Arthrodesis	20–25	— (joint fused)	3–5	15–25	Lower pain recurrence; adjacent joint overload possible
Suture Button Fixation	< 10	5–10	< 3	< 5	Minimal irritation; maintains motion; best for athletic RTP

Abbreviations: ORIF, open reduction and internal fixation; RTP, return to sports.

### 8.3. Future Direction

Ongoing research is exploring bioabsorbable magnesium and polymer‐based implants that may provide stable fixation without secondary removal procedures [47]. Intraoperative 3D navigation and augmented‐reality‐assisted reduction are emerging to enhance precision and reduce residual displacement [33]. Furthermore, AI‐driven diagnostic algorithms trained on WBCT and MRI datasets show early success in automatically detecting subtle Lisfranc malalignment and quantifying diastasis with near‐expert accuracy [21]. These innovations align with the translational mission of sports medicine and represent promising frontiers for improving both diagnostic accuracy and patient outcomes.

## 9. Conclusion

Lisfranc injuries are increasingly recognized due to advancements in imaging and growing clinical awareness, particularly among athletic populations. The LLC plays a critical role in midfoot stability, and its disruption can result in significant long‐term morbidity if not promptly identified and managed. Accurate diagnosis requires a high index of suspicion, a meticulous physical examination, and weight‐bearing imaging. Subtle or missed injuries can lead to chronic pain, deformity, and functional impairment. Optimal management depends on early diagnosis, assessment of stability, and anatomic reduction. Nonoperative treatment is reserved for nondisplaced injuries with intact joint alignment, while any evidence of instability or displacement warrants surgery. Surgical approach should be individualized, with fixation tailored to joint involvement and patient needs. Despite advances, delayed treatment and suboptimal fixation remain key risks for poor outcomes, underscoring the importance of timely, precise intervention. Further studies are needed to explore the best treatment option that optimizes outcomes at a low complication rate.

## Disclosure

All authors gave final approval of the article.

## Conflicts of Interest

The authors declare no conflicts of interest.

## Author Contributions

A.H. and J.A. conceived the study. Manuscript draft was written by A.H. and Y.A. J.A. contributed to critical review and revision.

## Funding

No funding was received for this manuscript.

## Data Availability

The authors have nothing to report.
